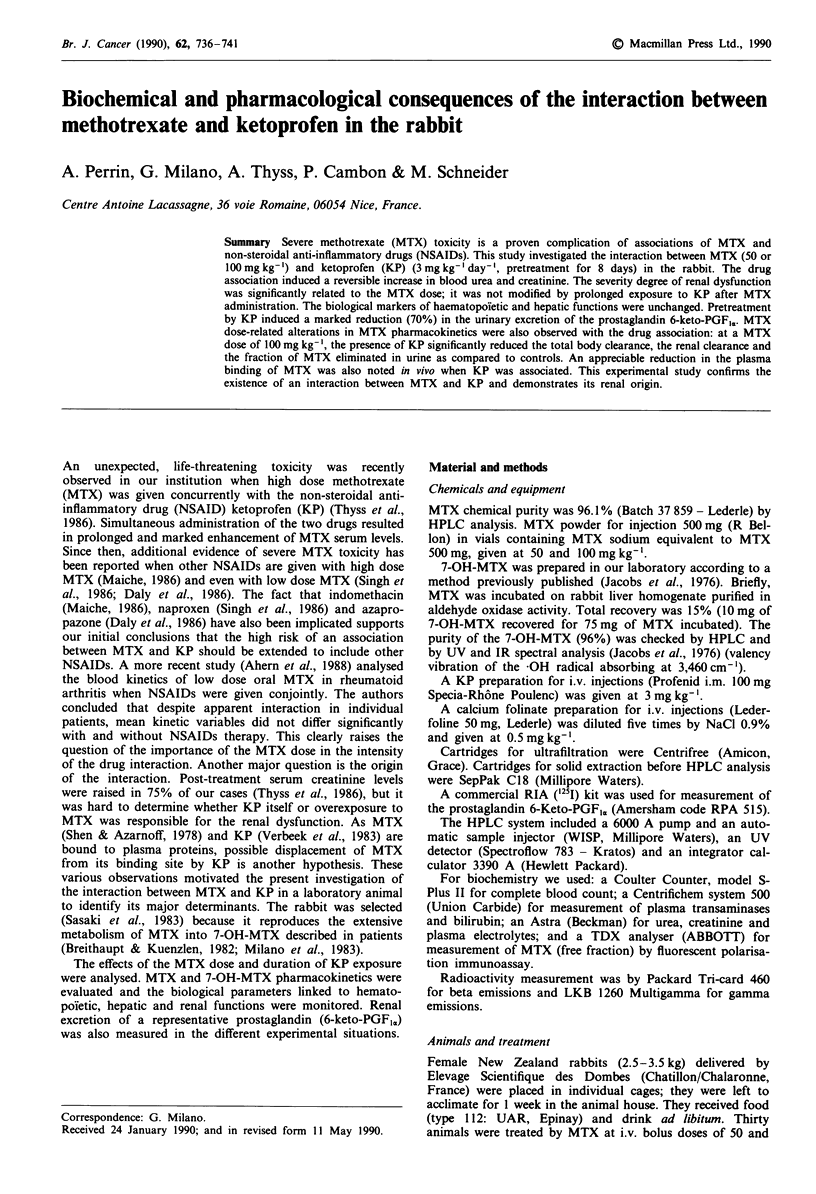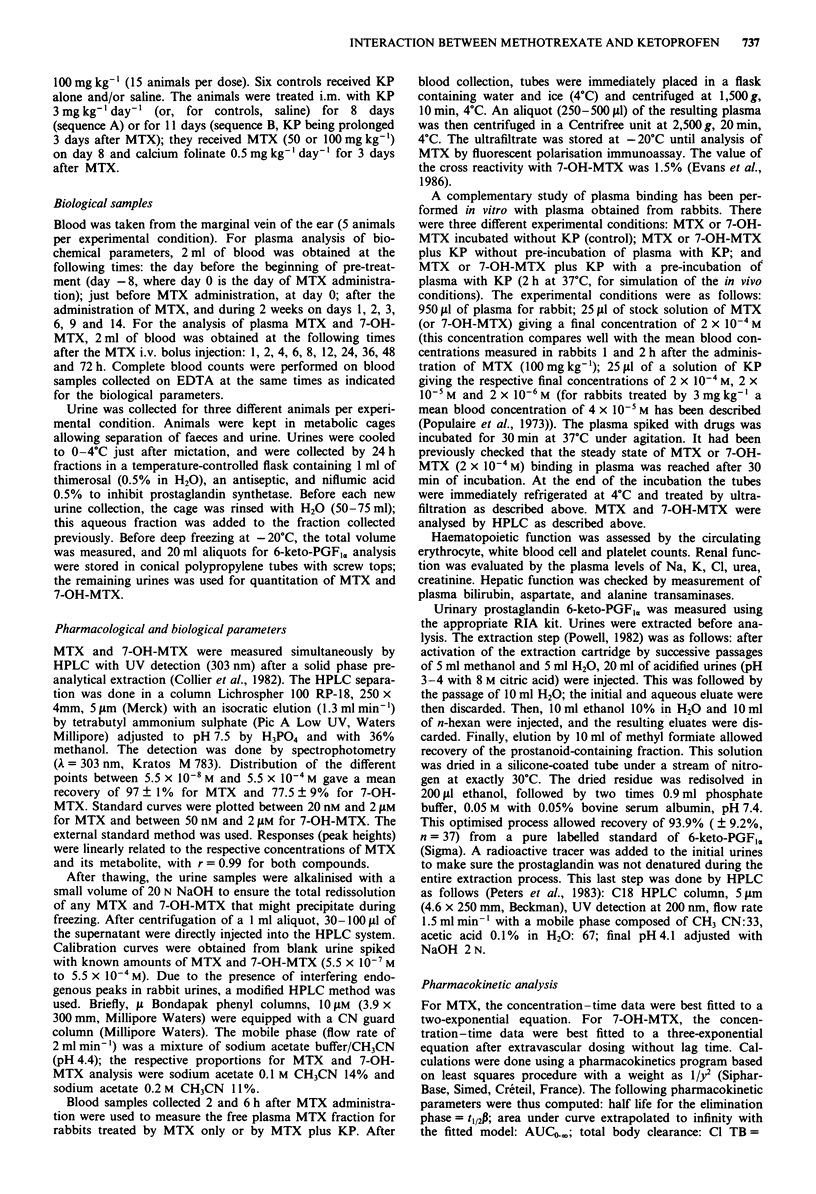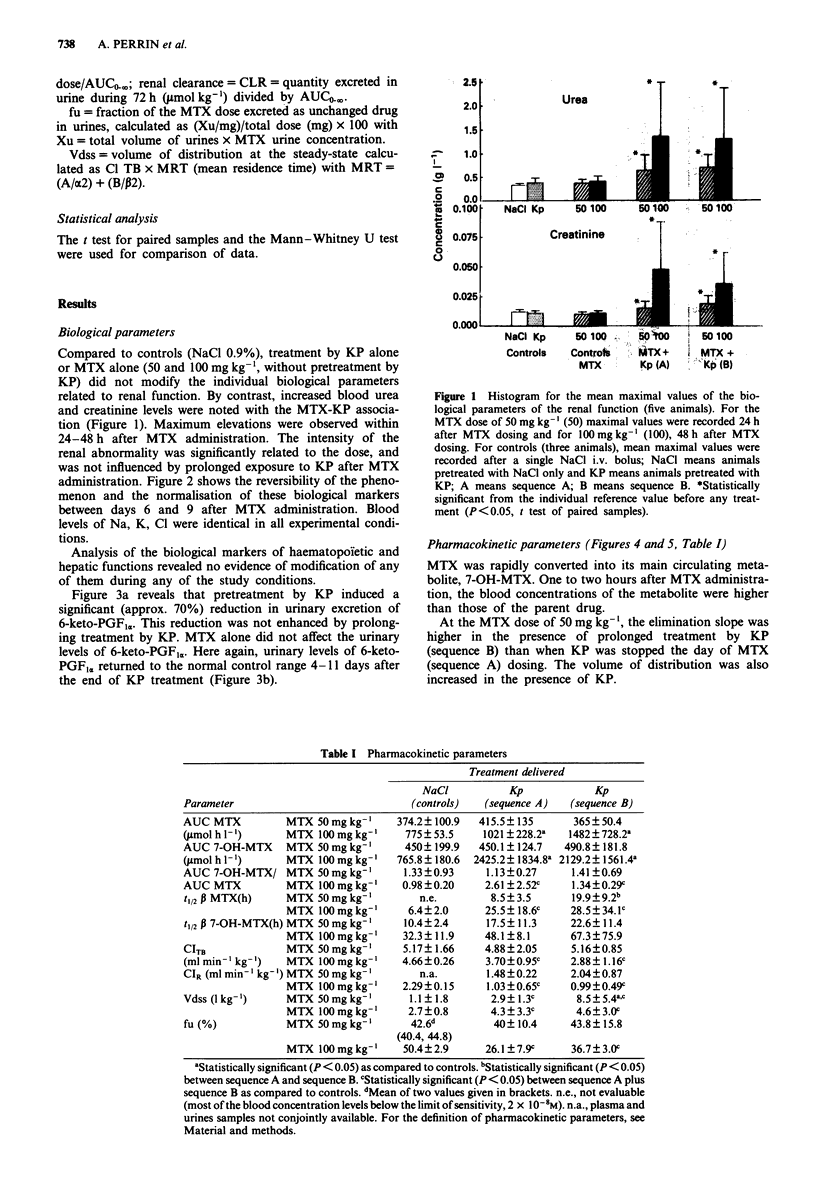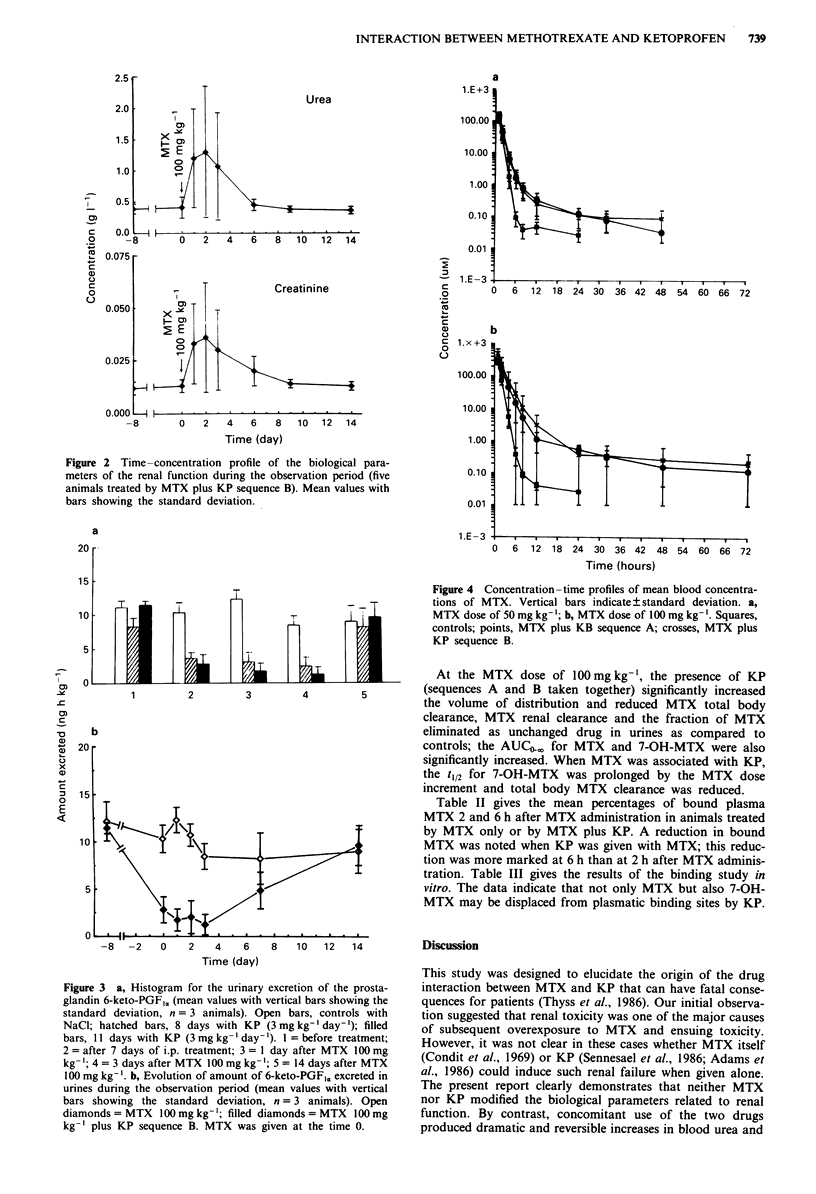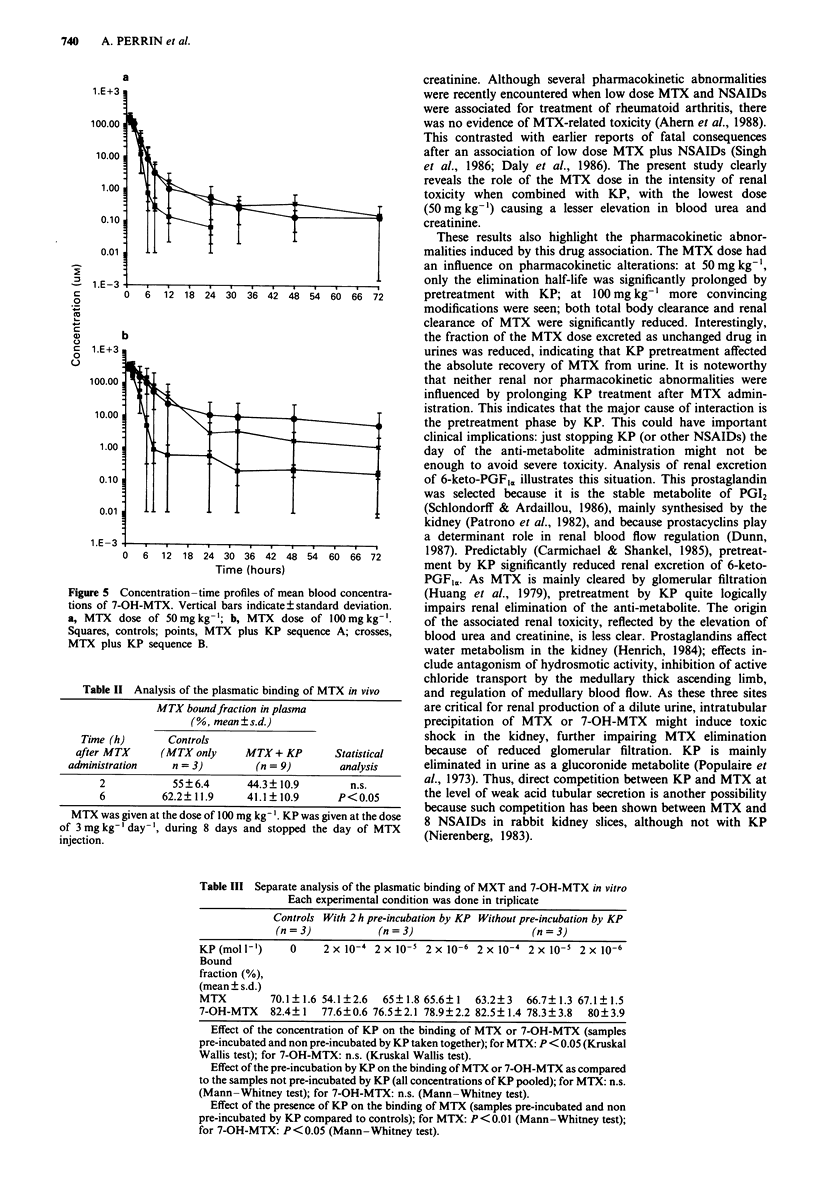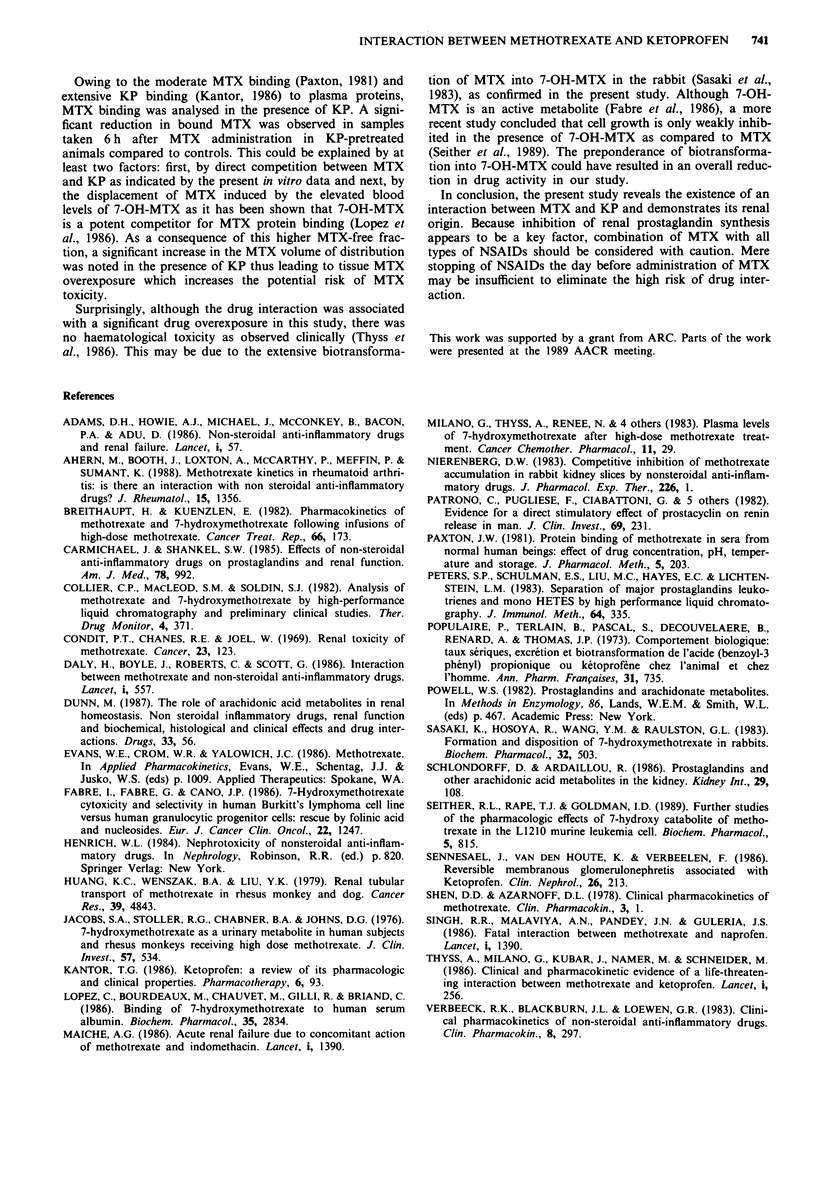# Biochemical and pharmacological consequences of the interaction between methotrexate and ketoprofen in the rabbit.

**DOI:** 10.1038/bjc.1990.369

**Published:** 1990-11

**Authors:** A. Perrin, G. Milano, A. Thyss, P. Cambon, M. Schneider

**Affiliations:** Centre Antoine Lacassagne, Nice, France.

## Abstract

Severe methotrexate (MTX) toxicity is a proven complication of associations of MTX and non-steroidal anti-inflammatory drugs (NSAIDs). This study investigated the interaction between MTX (50 or 100 mg kg-1) and ketoprofen (KP) (3 mg kg-1 day-1, pretreatment for 8 days) in the rabbit. The drug association induced a reversible increase in blood urea and creatinine. The severity degree of renal dysfunction was significantly related to the MTX dose; it was not modified by prolonged exposure to KP after MTX administration. The biological markers of haematopoietic and hepatic functions were unchanged. Pretreatment by KP induced a marked reduction (70%) in the urinary excretion of the prostaglandin 6-keto-PGF1 alpha. MTX dose-related alterations in MTX pharmacokinetics were also observed with the drug association: at a MTX dose of 100 mg kg-1, the presence of KP significantly reduced the total body clearance, the renal clearance and the fraction of MTX eliminated in urine as compared to controls. An appreciable reduction in the plasma binding of MTX was also noted in vivo when KP was associated. This experimental study confirms the existence of an interaction between MTX and KP and demonstrates its renal origin.


Br. J. Cancer (1990), 62, 736-741 © Macmillan Press Ltd., 1990

### Biochemical and pharmacological consequences of the interaction between methotrexate and ketoprofen in the rabbit

A. Perrin, G. Milano, A. Thyss, P. Cambon & M. Schneider

*Centre Antoine Lacassagne, 36 voie Romaine, 06054 Nice, France.*

**Summary** Severe methotrexate (MTX) toxicity is a proven complication of associations of MTX and non-steroidal anti-inflammatory drugs (NSAIDs). This study investigated the interaction between MTX (50 or 100 mg $kg^{-1}$) and ketoprofen (KP) (3 mg $kg^{-1}$ $day^{-1}$, pretreatment for 8 days) in the rabbit. The drug association induced a reversible increase in blood urea and creatinine. The severity degree of renal dysfunction was significantly related to the MTX dose; it was not modified by prolonged exposure to KP after MTX administration. The biological markers of haematopoïetic and hepatic functions were unchanged. Pretreatment by KP induced a marked reduction (70%) in the urinary excretion of the prostaglandin 6-keto-$PGF_{1\alpha}$. MTX dose-related alterations in MTX pharmacokinetics were also observed with the drug association: at a MTX dose of 100 mg $kg^{-1}$, the presence of KP significantly reduced the total body clearance, the renal clearance and the fraction of MTX eliminated in urine as compared to controls. An appreciable reduction in the plasma binding of MTX was also noted *in vivo* when KP was associated. This experimental study confirms the existence of an interaction between MTX and KP and demonstrates its renal origin.

An unexpected, life-threatening toxicity was recently observed in our institution when high dose methotrexate (MTX) was given concurrently with the non-steroidal anti-inflammatory drug (NSAID) ketoprofen (KP) (Thyss *et al.*, 1986). Simultaneous administration of the two drugs resulted in prolonged and marked enhancement of MTX serum levels. Since then, additional evidence of severe MTX toxicity has been reported when other NSAIDs are given with high dose MTX (Maiche, 1986) and even with low dose MTX (Singh *et al.*, 1986; Daly *et al.*, 1986). The fact that indomethacin (Maiche, 1986), naproxen (Singh *et al.*, 1986) and azapropazone (Daly *et al.*, 1986) have also been implicated supports our initial conclusions that the high risk of an association between MTX and KP should be extended to include other NSAIDs. A more recent study (Ahern *et al.*, 1988) analysed the blood kinetics of low dose oral MTX in rheumatoid arthritis when NSAIDs were given conjointly. The authors concluded that despite apparent interaction in individual patients, mean kinetic variables did not differ significantly with and without NSAIDs therapy. This clearly raises the question of the importance of the MTX dose in the intensity of the drug interaction. Another major question is the origin of the interaction. Post-treatment serum creatinine levels were raised in 75% of our cases (Thyss *et al.*, 1986), but it was hard to determine whether KP itself or overexposure to MTX was responsible for the renal dysfunction. As MTX (Shen & Azarnoff, 1978) and KP (Verbeek *et al.*, 1983) are bound to plasma proteins, possible displacement of MTX from its binding site by KP is another hypothesis. These various observations motivated the present investigation of the interaction between MTX and KP in a laboratory animal to identify its major determinants. The rabbit was selected (Sasaki *et al.*, 1983) because it reproduces the extensive metabolism of MTX into 7-OH-MTX described in patients (Breithaupt & Kuenzlen, 1982; Milano *et al.*, 1983).

The effects of the MTX dose and duration of KP exposure were analysed. MTX and 7-OH-MTX pharmacokinetics were evaluated and the biological parameters linked to hematopoïetic, hepatic and renal functions were monitored. Renal excretion of a representative prostaglandin (6-keto-$PGF_{1\alpha}$) was also measured in the different experimental situations.

Correspondence: G. Milano.
Received 24 January 1990; and in revised form 11 May 1990.

### Material and methods

### *Chemicals and equipment*

MTX chemical purity was 96.1% (Batch 37 859 - Lederle) by HPLC analysis. MTX powder for injection 500 mg (R Bellon) in vials containing MTX sodium equivalent to MTX 500 mg, given at 50 and 100 mg $kg^{-1}$.

7-OH-MTX was prepared in our laboratory according to a method previously published (Jacobs *et al.*, 1976). Briefly, MTX was incubated on rabbit liver homogenate purified in aldehyde oxidase activity. Total recovery was 15% (10 mg of 7-OH-MTX recovered for 75 mg of MTX incubated). The purity of the 7-OH-MTX (96%) was checked by HPLC and by UV and IR spectral analysis (Jacobs *et al.*, 1976) (valency vibration of the ·OH radical absorbing at 3,460 $cm^{-1}$).

A KP preparation for i.v. injections (Profenid i.m. 100 mg Specia-Rhône Poulenc) was given at 3 mg $kg^{-1}$.

A calcium folinate preparation for i.v. injections (Lederfoline 50 mg, Lederle) was diluted five times by NaCl 0.9% and given at 0.5 mg $kg^{-1}$.

Cartridges for ultrafiltration were Centrifree (Amicon, Grace). Cartridges for solid extraction before HPLC analysis were SepPak C18 (Millipore Waters).

A commercial RIA ($^{125}I$) kit was used for measurement of the prostaglandin 6-Keto-$PGF_{1\alpha}$ (Amersham code RPA 515).

The HPLC system included a 6000 A pump and an automatic sample injector (WISP, Millipore Waters), an UV detector (Spectroflow 783 - Kratos) and an integrator calculator 3390 A (Hewlett Packard).

For biochemistry we used: a Coulter Counter, model S-Plus II for complete blood count; a Centrifichem system 500 (Union Carbide) for measurement of plasma transaminases and bilirubin; an Astra (Beckman) for urea, creatinine and plasma electrolytes; and a TDX analyser (ABBOTT) for measurement of MTX (free fraction) by fluorescent polarisation immunoassay.

Radioactivity measurement was by Packard Tri-card 460 for beta emissions and LKB 1260 Multigamma for gamma emissions.

### *Animals and treatment*

Female New Zealand rabbits (2.5-3.5 kg) delivered by Elevage Scientifique des Dombes (Chatillon/Chalaronne, France) were placed in individual cages; they were left to acclimate for 1 week in the animal house. They received food (type 112: UAR, Epinay) and drink *ad libitum*. Thirty animals were treated by MTX at i.v. bolus doses of 50 and

100 mg $kg^{-1}$ (15 animals per dose). Six controls received KP alone and/or saline. The animals were treated i.m. with KP 3 mg $kg^{-1}$ $day^{-1}$ (or, for controls, saline) for 8 days (sequence A) or for 11 days (sequence B, KP being prolonged 3 days after MTX); they received MTX (50 or 100 mg $kg^{-1}$) on day 8 and calcium folinate 0.5 mg $kg^{-1}$ $day^{-1}$ for 3 days after MTX.

*Biological samples*

Blood was taken from the marginal vein of the ear (5 animals per experimental condition). For plasma analysis of biochemical parameters, 2 ml of blood was obtained at the following times: the day before the beginning of pre-treatment (day −8, where day 0 is the day of MTX administration); just before MTX administration, at day 0; after the administration of MTX, and during 2 weeks on days 1, 2, 3, 6, 9 and 14. For the analysis of plasma MTX and 7-OH-MTX, 2 ml of blood was obtained at the following times after the MTX i.v. bolus injection: 1, 2, 4, 6, 8, 12, 24, 36, 48 and 72 h. Complete blood counts were performed on blood samples collected on EDTA at the same times as indicated for the biological parameters.

Urine was collected for three different animals per experimental condition. Animals were kept in metabolic cages allowing separation of faeces and urine. Urines were cooled to 0–4°C just after mictation, and were collected by 24 h fractions in a temperature-controlled flask containing 1 ml of thimerosal (0.5% in $H_2O$), an antiseptic, and niflumic acid 0.5% to inhibit prostaglandin synthetase. Before each new urine collection, the cage was rinsed with $H_2O$ (50–75 ml); this aqueous fraction was added to the fraction collected previously. Before deep freezing at −20°C, the total volume was measured, and 20 ml aliquots for 6-keto-$PGF_{1\alpha}$ analysis were stored in conical polypropylene tubes with screw tops; the remaining urines was used for quantitation of MTX and 7-OH-MTX.

*Pharmacological and biological parameters*

MTX and 7-OH-MTX were measured simultaneously by HPLC with UV detection (303 nm) after a solid phase pre-analytical extraction (Collier *et al.*, 1982). The HPLC separation was done in a column Lichrospher 100 RP-18, 250 × 4mm, 5 μm (Merck) with an isocratic elution (1.3 ml $min^{-1}$) by tetrabutyl ammonium sulphate (Pic A Low UV, Waters Millipore) adjusted to pH 7.5 by $H_3PO_4$ and with 36% methanol. The detection was done by spectrophotometry ($\lambda$ = 303 nm, Kratos M 783). Distribution of the different points between $5.5 \times 10^{-8}$ M and $5.5 \times 10^{-4}$ M gave a mean recovery of 97 ± 1% for MTX and 77.5 ± 9% for 7-OH-MTX. Standard curves were plotted between 20 nM and 2 μM for MTX and between 50 nM and 2 μM for 7-OH-MTX. The external standard method was used. Responses (peak heights) were linearly related to the respective concentrations of MTX and its metabolite, with $r = 0.99$ for both compounds.

After thawing, the urine samples were alkalinised with a small volume of 20 N NaOH to ensure the total redissolution of any MTX and 7-OH-MTX that might precipitate during freezing. After centrifugation of a 1 ml aliquot, 30–100 μl of the supernatant were directly injected into the HPLC system. Calibration curves were obtained from blank urine spiked with known amounts of MTX and 7-OH-MTX ($5.5 \times 10^{-7}$ M to $5.5 \times 10^{-4}$ M). Due to the presence of interfering endogenous peaks in rabbit urines, a modified HPLC method was used. Briefly, μ Bondapak phenyl columns, 10 μM (3.9 × 300 mm, Millipore Waters) were equipped with a CN guard column (Millipore Waters). The mobile phase (flow rate of 2 ml $min^{-1}$) was a mixture of sodium acetate buffer/$CH_3CN$ (pH 4.4); the respective proportions for MTX and 7-OH-MTX analysis were sodium acetate 0.1 M $CH_3CN$ 14% and sodium acetate 0.2 M $CH_3CN$ 11%.

Blood samples collected 2 and 6 h after MTX administration were used to measure the free plasma MTX fraction for rabbits treated by MTX only or by MTX plus KP. After blood collection, tubes were immediately placed in a flask containing water and ice (4°C) and centrifuged at 1,500 *g*, 10 min, 4°C. An aliquot (250–500 μl) of the resulting plasma was then centrifuged in a Centrifree unit at 2,500 *g*, 20 min, 4°C. The ultrafiltrate was stored at −20°C until analysis of MTX by fluorescent polarisation immunoassay. The value of the cross reactivity with 7-OH-MTX was 1.5% (Evans *et al.*, 1986).

A complementary study of plasma binding has been performed *in vitro* with plasma obtained from rabbits. There were three different experimental conditions: MTX or 7-OH-MTX incubated without KP (control); MTX or 7-OH-MTX plus KP without pre-incubation of plasma with KP; and MTX or 7-OH-MTX plus KP with a pre-incubation of plasma with KP (2 h at 37°C, for simulation of the *in vivo* conditions). The experimental conditions were as follows: 950 μl of plasma for rabbit; 25 μl of stock solution of MTX (or 7-OH-MTX) giving a final concentration of $2 \times 10^{-4}$ M (this concentration compares well with the mean blood concentrations measured in rabbits 1 and 2 h after the administration of MTX (100 mg $kg^{-1}$); 25 μl of a solution of KP giving the respective final concentrations of $2 \times 10^{-4}$ M, $2 \times 10^{-5}$ M and $2 \times 10^{-6}$ M (for rabbits treated by 3 mg $kg^{-1}$ a mean blood concentration of $4 \times 10^{-5}$ M has been described (Populaire *et al.*, 1973)). The plasma spiked with drugs was incubated for 30 min at 37°C under agitation. It had been previously checked that the steady state of MTX or 7-OH-MTX ($2 \times 10^{-4}$ M) binding in plasma was reached after 30 min of incubation. At the end of the incubation the tubes were immediately refrigerated at 4°C and treated by ultrafiltration as described above. MTX and 7-OH-MTX were analysed by HPLC as described above.

Haematopoietic function was assessed by the circulating erythrocyte, white blood cell and platelet counts. Renal function was evaluated by the plasma levels of Na, K, Cl, urea, creatinine. Hepatic function was checked by measurement of plasma bilirubin, aspartate, and alanine transaminases.

Urinary prostaglandin 6-keto-$PGF_{1\alpha}$ was measured using the appropriate RIA kit. Urines were extracted before analysis. The extraction step (Powell, 1982) was as follows: after activation of the extraction cartridge by successive passages of 5 ml methanol and 5 ml $H_2O$, 20 ml of acidified urines (pH 3–4 with 8 M citric acid) were injected. This was followed by the passage of 10 ml $H_2O$; the initial and aqueous eluate were then discarded. Then, 10 ml ethanol 10% in $H_2O$ and 10 ml of *n*-hexan were injected, and the resulting eluates were discarded. Finally, elution by 10 ml of methyl formiate allowed recovery of the prostanoid-containing fraction. This solution was dried in a silicone-coated tube under a stream of nitrogen at exactly 30°C. The dried residue was redisolved in 200 μl ethanol, followed by two times 0.9 ml phosphate buffer, 0.05 M with 0.05% bovine serum albumin, pH 7.4. This optimised process allowed recovery of 93.9% (± 9.2%, $n = 37$) from a pure labelled standard of 6-keto-$PGF_{1\alpha}$ (Sigma). A radioactive tracer was added to the initial urines to make sure the prostaglandin was not denatured during the entire extraction process. This last step was done by HPLC as follows (Peters *et al.*, 1983): C18 HPLC column, 5 μm (4.6 × 250 mm, Beckman), UV detection at 200 nm, flow rate 1.5 ml $min^{-1}$ with a mobile phase composed of $CH_3$ CN:33, acetic acid 0.1% in $H_2O$: 67; final pH 4.1 adjusted with NaOH 2 N.

*Pharmacokinetic analysis*

For MTX, the concentration–time data were best fitted to a two-exponential equation. For 7-OH-MTX, the concentration–time data were best fitted to a three-exponential equation after extravascular dosing without lag time. Calculations were done using a pharmacokinetics program based on least squares procedure with a weight as $1/y^2$ (Siphar-Base, Simed, Créteil, France). The following pharmacokinetic parameters were thus computed: half life for the elimination phase = $t_{1/2}\beta$; area under curve extrapolated to infinity with the fitted model: $AUC_{0-\infty}$; total body clearance: Cl TB =

dose/$AUC_{0-\infty}$; renal clearance = CLR = quantity excreted in urine during 72 h ($\mu$mol $kg^{-1}$) divided by $AUC_{0-\infty}$.

fu = fraction of the MTX dose excreted as unchanged drug in urines, calculated as (Xu/mg)/total dose (mg) × 100 with Xu = total volume of urines × MTX urine concentration.

Vdss = volume of distribution at the steady-state calculated as Cl TB × MRT (mean residence time) with MRT = $(A/\alpha 2) + (B/\beta 2)$.

### *Statistical analysis*

The *t* test for paired samples and the Mann–Whitney U test were used for comparison of data.

### Results

### *Biological parameters*

Compared to controls (NaCl 0.9%), treatment by KP alone or MTX alone (50 and 100 mg $kg^{-1}$, without pretreatment by KP) did not modify the individual biological parameters related to renal function. By contrast, increased blood urea and creatinine levels were noted with the MTX-KP association (Figure 1). Maximum elevations were observed within 24–48 h after MTX administration. The intensity of the renal abnormality was significantly related to the dose, and was not influenced by prolonged exposure to KP after MTX administration. Figure 2 shows the reversibility of the phenomenon and the normalisation of these biological markers between days 6 and 9 after MTX administration. Blood levels of Na, K, Cl were identical in all experimental conditions.

Analysis of the biological markers of haematopoïetic and hepatic functions revealed no evidence of modification of any of them during any of the study conditions.

Figure 3a reveals that pretreatment by KP induced a significant (approx. 70%) reduction in urinary excretion of 6-keto-$PGF_{1\alpha}$. This reduction was not enhanced by prolonging treatment by KP. MTX alone did not affect the urinary levels of 6-keto-$PGF_{1\alpha}$. Here again, urinary levels of 6-keto-$PGF_{1\alpha}$ returned to the normal control range 4–11 days after the end of KP treatment (Figure 3b).

**Figure 1** Histogram for the mean maximal values of the biological parameters of the renal function (five animals). For the MTX dose of 50 mg $kg^{-1}$ (50) maximal values were recorded 24 h after MTX dosing and for 100 mg $kg^{-1}$ (100), 48 h after MTX dosing. For controls (three animals), mean maximal values were recorded after a single NaCl i.v. bolus; NaCl means animals pretreated with NaCl only and KP means animals pretreated with KP; A means sequence A; B means sequence B. *Statistically significant from the individual reference value before any treatment ($P < 0.05$, *t* test of paired samples).

### *Pharmacokinetic parameters (Figures 4 and 5, Table I)*

MTX was rapidly converted into its main circulating metabolite, 7-OH-MTX. One to two hours after MTX administration, the blood concentrations of the metabolite were higher than those of the parent drug.

At the MTX dose of 50 mg $kg^{-1}$, the elimination slope was higher in the presence of prolonged treatment by KP (sequence B) than when KP was stopped the day of MTX (sequence A) dosing. The volume of distribution was also increased in the presence of KP.

**Table I** Pharmacokinetic parameters

| Parameter | | Treatment delivered: NaCl (controls) | Kp (sequence A) | Kp (sequence B) |
|---|---|---|---|---|
| AUC MTX | MTX 50 mg $kg^{-1}$ | 374.2 ± 100.9 | 415.5 ± 135 | 365 ± 50.4 |
| ($\mu$mol h $l^{-1}$) | MTX 100 mg $kg^{-1}$ | 775 ± 53.5 | 1021 ± 228.2[a] | 1482 ± 728.2[a] |
| AUC 7-OH-MTX | MTX 50 mg $kg^{-1}$ | 450 ± 199.9 | 450.1 ± 124.7 | 490.8 ± 181.8 |
| ($\mu$mol h $l^{-1}$) | MTX 100 mg $kg^{-1}$ | 765.8 ± 180.6 | 2425.2 ± 1834.8[a] | 2129.2 ± 1561.4[a] |
| AUC 7-OH-MTX/ | MTX 50 mg $kg^{-1}$ | 1.33 ± 0.93 | 1.13 ± 0.27 | 1.41 ± 0.69 |
| AUC MTX | MTX 100 mg $kg^{-1}$ | 0.98 ± 0.20 | 2.61 ± 2.52[c] | 1.34 ± 0.29[c] |
| $t_{1/2}\ \beta$ MTX(h) | MTX 50 mg $kg^{-1}$ | n.e. | 8.5 ± 3.5 | 19.9 ± 9.2[b] |
| | MTX 100 mg $kg^{-1}$ | 6.4 ± 2.0 | 25.5 ± 18.6[c] | 28.5 ± 34.1[c] |
| $t_{1/2}\ \beta$ 7-OH-MTX(h) | MTX 50 mg $kg^{-1}$ | 10.4 ± 2.4 | 17.5 ± 11.3 | 22.6 ± 11.4 |
| | MTX 100 mg $kg^{-1}$ | 32.3 ± 11.9 | 48.1 ± 8.1 | 67.3 ± 75.9 |
| $CI_{TB}$ | MTX 50 mg $kg^{-1}$ | 5.17 ± 1.66 | 4.88 ± 2.05 | 5.16 ± 0.85 |
| (ml $min^{-1}$ $kg^{-1}$) | MTX 100 mg $kg^{-1}$ | 4.66 ± 0.26 | 3.70 ± 0.95[c] | 2.88 ± 1.16[c] |
| $CI_R$ (ml $min^{-1}$ $kg^{-1}$) | MTX 50 mg $kg^{-1}$ | n.a. | 1.48 ± 0.22 | 2.04 ± 0.87 |
| | MTX 100 mg $kg^{-1}$ | 2.29 ± 0.15 | 1.03 ± 0.65[c] | 0.99 ± 0.49[c] |
| Vdss (l $kg^{-1}$) | MTX 50 mg $kg^{-1}$ | 1.1 ± 1.8 | 2.9 ± 1.3[c] | 8.5 ± 5.4[a,c] |
| | MTX 100 mg $kg^{-1}$ | 2.7 ± 0.8 | 4.3 ± 3.3[c] | 4.6 ± 3.0[c] |
| fu (%) | MTX 50 mg $kg^{-1}$ | 42.6[d] (40.4, 44.8) | 40 ± 10.4 | 43.8 ± 15.8 |
| | MTX 100 mg $kg^{-1}$ | 50.4 ± 2.9 | 26.1 ± 7.9[c] | 36.7 ± 3.0[c] |

[a]Statistically significant ($P < 0.05$) as compared to controls. [b]Statistically significant ($P < 0.05$) between sequence A and sequence B. [c]Statistically significant ($P < 0.05$) between sequence A plus sequence B as compared to controls. [d]Mean of two values given in brackets. n.e., not evaluable (most of the blood concentration levels below the limit of sensitivity, $2 \times 10^{-8}$M). n.a., plasma and urines samples not conjointly available. For the definition of pharmacokinetic parameters, see Material and methods.

**Figure 2** Time–concentration profile of the biological parameters of the renal function during the observation period (five animals treated by MTX plus KP sequence B). Mean values with bars showing the standard deviation.

**Figure 3** **a**, Histogram for the urinary excretion of the prostaglandin 6-keto-PGF$_{1\alpha}$ (mean values with vertical bars showing the standard deviation, $n = 3$ animals). Open bars, controls with NaCl; hatched bars, 8 days with KP (3 mg kg$^{-1}$ day$^{-1}$); filled bars, 11 days with KP (3 mg kg$^{-1}$ day$^{-1}$). 1 = before treatment; 2 = after 7 days of i.p. treatment; 3 = 1 day after MTX 100 mg kg$^{-1}$; 4 = 3 days after MTX 100 mg kg$^{-1}$; 5 = 14 days after MTX 100 mg kg$^{-1}$. **b**, Evolution of amount of 6-keto-PGF$_{1\alpha}$ excreted in urines during the observation period (mean values with vertical bars showing the standard deviation, $n = 3$ animals). Open diamonds = MTX 100 mg kg$^{-1}$; filled diamonds = MTX 100 mg kg$^{-1}$ plus KP sequence B. MTX was given at the time 0.

**Figure 4** Concentration–time profiles of mean blood concentrations of MTX. Vertical bars indicate ± standard deviation. **a**, MTX dose of 50 mg kg$^{-1}$; **b**, MTX dose of 100 mg kg$^{-1}$. Squares, controls; points, MTX plus KB sequence A; crosses, MTX plus KP sequence B.

At the MTX dose of 100 mg kg$^{-1}$, the presence of KP (sequences A and B taken together) significantly increased the volume of distribution and reduced MTX total body clearance, MTX renal clearance and the fraction of MTX eliminated as unchanged drug in urines as compared to controls; the $AUC_{0-\infty}$ for MTX and 7-OH-MTX were also significantly increased. When MTX was associated with KP, the $t_{1/2}$ for 7-OH-MTX was prolonged by the MTX dose increment and total body MTX clearance was reduced.

Table II gives the mean percentages of bound plasma MTX 2 and 6 h after MTX administration in animals treated by MTX only or by MTX plus KP. A reduction in bound MTX was noted when KP was given with MTX; this reduction was more marked at 6 h than at 2 h after MTX administration. Table III gives the results of the binding study *in vitro*. The data indicate that not only MTX but also 7-OH-MTX may be displaced from plasmatic binding sites by KP.

### Discussion

This study was designed to elucidate the origin of the drug interaction between MTX and KP that can have fatal consequences for patients (Thyss *et al.*, 1986). Our initial observation suggested that renal toxicity was one of the major causes of subsequent overexposure to MTX and ensuing toxicity. However, it was not clear in these cases whether MTX itself (Condit *et al.*, 1969) or KP (Sennesael *et al.*, 1986; Adams *et al.*, 1986) could induce such renal failure when given alone. The present report clearly demonstrates that neither MTX nor KP modified the biological parameters related to renal function. By contrast, concomitant use of the two drugs produced dramatic and reversible increases in blood urea and

**Figure 5** Concentration–time profiles of mean blood concentrations of 7-OH-MTX. Vertical bars indicate ± standard deviation. **a**, MTX dose of 50 mg $kg^{-1}$; **b**, MTX dose of 100 mg $kg^{-1}$. Squares, controls; points, MTX plus KP sequence A; crosses, MTX plus KP sequence B.

**Table II** Analysis of the plasmatic binding of MTX *in vivo*

| *Time (h) after MTX administration* | *MTX bound fraction in plasma (%, mean ± s.d.)* Controls (MTX only n = 3) | *MTX + KP (n = 9)* | *Statistical analysis* |
|---|---|---|---|
| 2 | 55 ± 6.4 | 44.3 ± 10.9 | n.s. |
| 6 | 62.2 ± 11.9 | 41.1 ± 10.9 | $P < 0.05$ |

MTX was given at the dose of 100 mg $kg^{-1}$. KP was given at the dose of 3 mg $kg^{-1}$ $day^{-1}$, during 8 days and stopped the day of MTX injection.

creatinine. Although several pharmacokinetic abnormalities were recently encountered when low dose MTX and NSAIDs were associated for treatment of rheumatoid arthritis, there was no evidence of MTX-related toxicity (Ahern *et al.*, 1988). This contrasted with earlier reports of fatal consequences after an association of low dose MTX plus NSAIDs (Singh *et al.*, 1986; Daly *et al.*, 1986). The present study clearly reveals the role of the MTX dose in the intensity of renal toxicity when combined with KP, with the lowest dose (50 mg $kg^{-1}$) causing a lesser elevation in blood urea and creatinine.

These results also highlight the pharmacokinetic abnormalities induced by this drug association. The MTX dose had an influence on pharmacokinetic alterations: at 50 mg $kg^{-1}$, only the elimination half-life was significantly prolonged by pretreatment with KP; at 100 mg $kg^{-1}$ more convincing modifications were seen; both total body clearance and renal clearance of MTX were significantly reduced. Interestingly, the fraction of the MTX dose excreted as unchanged drug in urines was reduced, indicating that KP pretreatment affected the absolute recovery of MTX from urine. It is noteworthy that neither renal nor pharmacokinetic abnormalities were influenced by prolonging KP treatment after MTX administration. This indicates that the major cause of interaction is the pretreatment phase by KP. This could have important clinical implications: just stopping KP (or other NSAIDs) the day of the anti-metabolite administration might not be enough to avoid severe toxicity. Analysis of renal excretion of 6-keto-$PGF_{1\alpha}$ illustrates this situation. This prostaglandin was selected because it is the stable metabolite of $PGI_2$ (Schlondorff & Ardaillou, 1986), mainly synthesised by the kidney (Patrono *et al.*, 1982), and because prostacyclins play a determinant role in renal blood flow regulation (Dunn, 1987). Predictably (Carmichael & Shankel, 1985), pretreatment by KP significantly reduced renal excretion of 6-keto-$PGF_{1\alpha}$. As MTX is mainly cleared by glomerular filtration (Huang *et al.*, 1979), pretreatment by KP quite logically impairs renal elimination of the anti-metabolite. The origin of the associated renal toxicity, reflected by the elevation of blood urea and creatinine, is less clear. Prostaglandins affect water metabolism in the kidney (Henrich, 1984); effects include antagonism of hydrosmotic activity, inhibition of active chloride transport by the medullary thick ascending limb, and regulation of medullary blood flow. As these three sites are critical for renal production of a dilute urine, intratubular precipitation of MTX or 7-OH-MTX might induce toxic shock in the kidney, further impairing MTX elimination because of reduced glomerular filtration. KP is mainly eliminated in urine as a glucoronide metabolite (Populaire *et al.*, 1973). Thus, direct competition between KP and MTX at the level of weak acid tubular secretion is another possibility because such competition has been shown between MTX and 8 NSAIDs in rabbit kidney slices, although not with KP (Nierenberg, 1983).

**Table III** Separate analysis of the plasmatic binding of MXT and 7-OH-MTX *in vitro*
Each experimental condition was done in triplicate

| | *Controls (n = 3)* | *With 2 h pre-incubation by KP (n = 3)* | | | *Without pre-incubation by KP (n = 3)* | | |
|---|---|---|---|---|---|---|---|
| KP (mol $l^{-1}$) | 0 | $2 \times 10^{-4}$ | $2 \times 10^{-5}$ | $2 \times 10^{-6}$ | $2 \times 10^{-4}$ | $2 \times 10^{-5}$ | $2 \times 10^{-6}$ |
| Bound fraction (%), (mean ± s.d.) | | | | | | | |
| MTX | 70.1 ± 1.6 | 54.1 ± 2.6 | 65 ± 1.8 | 65.6 ± 1 | 63.2 ± 3 | 66.7 ± 1.3 | 67.1 ± 1.5 |
| 7-OH-MTX | 82.4 ± 1 | 77.6 ± 0.6 | 76.5 ± 2.1 | 78.9 ± 2.2 | 82.5 ± 1.4 | 78.3 ± 3.8 | 80 ± 3.9 |

Effect of the concentration of KP on the binding of MTX or 7-OH-MTX (samples pre-incubated and non pre-incubated by KP taken together); for MTX: $P < 0.05$ (Kruskal Wallis test); for 7-OH-MTX: n.s. (Kruskal Wallis test).

Effect of the pre-incubation by KP on the binding of MTX or 7-OH-MTX as compared to the samples not pre-incubated by KP (all concentrations of KP pooled); for MTX: n.s. (Mann–Whitney test); for 7-OH-MTX: n.s. (Mann–Whitney test).

Effect of the presence of KP on the binding of MTX (samples pre-incubated and non pre-incubated by KP compared to controls); for MTX: $P < 0.01$ (Mann–Whitney test); for 7-OH-MTX: $P < 0.05$ (Mann–Whitney test).

Owing to the moderate MTX binding (Paxton, 1981) and extensive KP binding (Kantor, 1986) to plasma proteins, MTX binding was analysed in the presence of KP. A significant reduction in bound MTX was observed in samples taken 6 h after MTX administration in KP-pretreated animals compared to controls. This could be explained by at least two factors: first, by direct competition between MTX and KP as indicated by the present *in vitro* data and next, by the displacement of MTX induced by the elevated blood levels of 7-OH-MTX as it has been shown that 7-OH-MTX is a potent competitor for MTX protein binding (Lopez *et al.*, 1986). As a consequence of this higher MTX-free fraction, a significant increase in the MTX volume of distribution was noted in the presence of KP thus leading to tissue MTX overexposure which increases the potential risk of MTX toxicity.

Surprisingly, although the drug interaction was associated with a significant drug overexposure in this study, there was no haematological toxicity as observed clinically (Thyss *et al.*, 1986). This may be due to the extensive biotransformation of MTX into 7-OH-MTX in the rabbit (Sasaki *et al.*, 1983), as confirmed in the present study. Although 7-OH-MTX is an active metabolite (Fabre *et al.*, 1986), a more recent study concluded that cell growth is only weakly inhibited in the presence of 7-OH-MTX as compared to MTX (Seither *et al.*, 1989). The preponderance of biotransformation into 7-OH-MTX could have resulted in an overall reduction in drug activity in our study.

In conclusion, the present study reveals the existence of an interaction between MTX and KP and demonstrates its renal origin. Because inhibition of renal prostaglandin synthesis appears to be a key factor, combination of MTX with all types of NSAIDs should be considered with caution. Mere stopping of NSAIDs the day before administration of MTX may be insufficient to eliminate the high risk of drug interaction.

This work was supported by a grant from ARC. Parts of the work were presented at the 1989 AACR meeting.

### References

ADAMS, D.H., HOWIE, A.J., MICHAEL, J., MCCONKEY, B., BACON, P.A. & ADU, D. (1986). Non-steroidal anti-inflammatory drugs and renal failure. *Lancet*, **i**, 57.

AHERN, M., BOOTH, J., LOXTON, A., MCCARTHY, P., MEFFIN, P. & SUMANT, K. (1988). Methotrexate kinetics in rheumatoid arthritis: is there an interaction with non steroidal anti-inflammatory drugs? *J. Rheumatol.*, **15**, 1356.

BREITHAUPT, H. & KUENZLEN, E. (1982). Pharmacokinetics of methotrexate and 7-hydroxymethotrexate following infusions of high-dose methotrexate. *Cancer Treat. Rep.*, **66**, 173.

CARMICHAEL, J. & SHANKEL, S.W. (1985). Effects of non-steroidal anti-inflammatory drugs on prostaglandins and renal function. *Am. J. Med.*, **78**, 992.

COLLIER, C.P., MACLEOD, S.M. & SOLDIN, S.J. (1982). Analysis of methotrexate and 7-hydroxymethotrexate by high-performance liquid chromatography and preliminary clinical studies. *Ther. Drug Monitor*, **4**, 371.

CONDIT, P.T., CHANES, R.E. & JOEL, W. (1969). Renal toxicity of methotrexate. *Cancer*, **23**, 123.

DALY, H., BOYLE, J., ROBERTS, C. & SCOTT, G. (1986). Interaction between methotrexate and non-steroidal anti-inflammatory drugs. *Lancet*, **i**, 557.

DUNN, M. (1987). The role of arachidonic acid metabolites in renal homeostasis. Non steroidal inflammatory drugs, renal function and biochemical, histological and clinical effects and drug interactions. *Drugs*, **33**, 56.

EVANS, W.E., CROM, W.R. & YALOWICH, J.C. (1986). Methotrexate. In *Applied Pharmacokinetics*, Evans, W.E., Schentag, J.J. & Jusko, W.S. (eds) p. 1009. Applied Therapeutics: Spokane, WA.

FABRE, I., FABRE, G. & CANO, J.P. (1986). 7-Hydroxymethotrexate cytoxicity and selectivity in human Burkitt's lymphoma cell line versus human granulocytic progenitor cells: rescue by folinic acid and nucleosides. *Eur. J. Cancer Clin. Oncol.*, **22**, 1247.

HENRICH, W.L. (1984). Nephrotoxicity of nonsteroidal anti-inflammatory drugs. In *Nephrology*, Robinson, R.R. (ed.) p. 820. Springer Verlag: New York.

HUANG, K.C., WENSZAK, B.A. & LIU, Y.K. (1979). Renal tubular transport of methotrexate in rhesus monkey and dog. *Cancer Res.*, **39**, 4843.

JACOBS, S.A., STOLLER, R.G., CHABNER, B.A. & JOHNS, D.G. (1976). 7-hydroxymethotrexate as a urinary metabolite in human subjects and rhesus monkeys receiving high dose methotrexate. *J. Clin. Invest.*, **57**, 534.

KANTOR, T.G. (1986). Ketoprofen: a review of its pharmacologic and clinical properties. *Pharmacotherapy*, **6**, 93.

LOPEZ, C., BOURDEAUX, M., CHAUVET, M., GILLI, R. & BRIAND, C. (1986). Binding of 7-hydroxymethotrexate to human serum albumin. *Biochem. Pharmacol.*, **35**, 2834.

MAICHE, A.G. (1986). Acute renal failure due to concomitant action of methotrexate and indomethacin. *Lancet*, **i**, 1390.

MILANO, G., THYSS, A., RENEE, N. & 4 others (1983). Plasma levels of 7-hydroxymethotrexate after high-dose methotrexate treatment. *Cancer Chemother. Pharmacol.*, **11**, 29.

NIERENBERG, D.W. (1983). Competitive inhibition of methotrexate accumulation in rabbit kidney slices by nonsteroidal anti-inflammatory drugs. *J. Pharmacol. Exp. Ther.*, **226**, 1.

PATRONO, C., PUGLIESE, F., CIABATTONI, G. & 5 others (1982). Evidence for a direct stimulatory effect of prostacyclin on renin release in man. *J. Clin. Invest.*, **69**, 231.

PAXTON, J.W. (1981). Protein binding of methotrexate in sera from normal human beings: effect of drug concentration, pH, temperature and storage. *J. Pharmacol. Meth.*, **5**, 203.

PETERS, S.P., SCHULMAN, E.S., LIU, M.C., HAYES, E.C. & LICHTENSTEIN, L.M. (1983). Separation of major prostaglandins leukotrienes and mono HETES by high performance liquid chromatography. *J. Immunol. Meth.*, **64**, 335.

POPULAIRE, P., TERLAIN, B., PASCAL, S., DECOUVELAERE, B., RENARD, A. & THOMAS, J.P. (1973). Comportement biologique: taux sériques, excrétion et biotransformation de l'acide (benzoyl-3 phényl) propionique ou kétoprofène chez l'animal et chez l'homme. *Ann. Pharm. Françaises*, **31**, 735.

POWELL, W.S. (1982). Prostaglandins and arachidonate metabolites. In *Methods in Enzymology, 86*, Lands, W.E.M. & Smith, W.L. (eds) p. 467. Academic Press: New York.

SASAKI, K., HOSOYA, R., WANG, Y.M. & RAULSTON, G.L. (1983). Formation and disposition of 7-hydroxymethotrexate in rabbits. *Biochem. Pharmacol.*, **32**, 503.

SCHLONDORFF, D. & ARDAILLOU, R. (1986). Prostaglandins and other arachidonic acid metabolites in the kidney. *Kidney Int.*, **29**, 108.

SEITHER, R.L., RAPE, T.J. & GOLDMAN, I.D. (1989). Further studies of the pharmacologic effects of 7-hydroxy catabolite of methotrexate in the L1210 murine leukemia cell. *Biochem. Pharmacol.*, **5**, 815.

SENNESAEL, J., VAN DEN HOUTE, K. & VERBEELEN, F. (1986). Reversible membranous glomerulonephretis associated with Ketoprofen. *Clin. Nephrol.*, **26**, 213.

SHEN, D.D. & AZARNOFF, D.L. (1978). Clinical pharmacokinetics of methotrexate. *Clin. Pharmacokin.*, **3**, 1.

SINGH, R.R., MALAVIYA, A.N., PANDEY, J.N. & GULERIA, J.S. (1986). Fatal interaction between methotrexate and naprofen. *Lancet*, **i**, 1390.

THYSS, A., MILANO, G., KUBAR, J., NAMER, M. & SCHNEIDER, M. (1986). Clinical and pharmacokinetic evidence of a life-threatening interaction between methotrexate and ketoprofen. *Lancet*, **i**, 256.

VERBEECK, R.K., BLACKBURN, J.L. & LOEWEN, G.R. (1983). Clinical pharmacokinetics of non-steroidal anti-inflammatory drugs. *Clin. Pharmacokin.*, **8**, 297.